# Electric Pulse Stimulation of Cultured Murine Muscle Cells Reproduces Gene Expression Changes of Trained Mouse Muscle

**DOI:** 10.1371/journal.pone.0010970

**Published:** 2010-06-04

**Authors:** Nathalie Burch, Anne-Sophie Arnold, Flurin Item, Serge Summermatter, Gesa Brochmann Santana Santos, Martine Christe, Urs Boutellier, Marco Toigo, Christoph Handschin

**Affiliations:** 1 Institute of Physiology and Zurich Center for Integrative Human Physiology (ZIHP), University of Zurich, Zurich, Switzerland; 2 Exercise Physiology, Institute of Human Movement Sciences, ETH Zurich, Zurich, Switzerland; 3 Biozentrum, Department of Pharmacology/Neurobiology, University of Basel, Basel, Switzerland; University Hospital Vall d'Hebron, Spain

## Abstract

Adequate levels of physical activity are at the center of a healthy lifestyle. However, the molecular mechanisms that mediate the beneficial effects of exercise remain enigmatic. This gap in knowledge is caused by the lack of an amenable experimental model system. Therefore, we optimized electric pulse stimulation of muscle cells to closely recapitulate the plastic changes in gene expression observed in a trained skeletal muscle. The exact experimental conditions were established using the peroxisome proliferator-activated receptor γ coactivator 1α (PGC-1α) as a marker for an endurance-trained muscle fiber. We subsequently compared the changes in the relative expression of metabolic and myofibrillar genes in the muscle cell system with those observed in mouse muscle *in vivo* following either an acute or repeated bouts of treadmill exercise. Importantly, in electrically stimulated C2C12 mouse muscle cells, the qualitative transcriptional adaptations were almost identical to those in trained muscle, but differ from the acute effects of exercise on muscle gene expression. In addition, significant alterations in the expression of myofibrillar proteins indicate that this stimulation could be used to modulate the fiber-type of muscle cells in culture. Our data thus describe an experimental cell culture model for the study of at least some of the transcriptional aspects of skeletal muscle adaptation to physical activity. This system will be useful for the study of the molecular mechanisms that regulate exercise adaptation in muscle.

## Introduction

Physical activity results in a number of phenotypic adaptations of skeletal muscle [1], [2]. If performed repeatedly, exercise thereby confers health benefits by preventing and ameliorating a number of chronic diseases, improving the quality of life and increasing life expectancy [3]. Intriguingly though, the molecular mechanisms that underlie the corresponding plastic changes of muscle fibers are poorly defined. Progress in understanding these mechanisms would be facilitated by an experimentally amenable cell culture model. Electrical and mechanical signals, e.g. motor neuron activity and muscle fiber stretch, are two of the major exercise-associated stimuli that result in a remodeling of muscle fibers. Motor neuron activation of muscle fibers can be replicated by electric pulse stimulation (EPS) of muscle myotubes in culture. For example, electrical stimulation of muscle cells in culture increases contractile properties [4] and accelerates sarcomere assembly [5]. Furthermore, EPS-induced changes in gene expression patterns and metabolic properties have been reported [6], [7], [8]. Unfortunately, the effects of electric stimulation on the development and function of cultured muscle cells remain controversial and vary between different muscle cell types and stimulation conditions [9], [10], [11], [12].

To study exercise effects in an experimental cultured muscle fiber model, the gene expression changes triggered by exercise *in vivo* and the modulation of transcriptional activity of cultured, EPS-stimulated muscle cells have to be compared. Obviously though, *in vivo*, these changes differ dramatically between the acute response to a single bout of exercise and the chronic effects of training on muscle, with the latter conferring most of the health benefits [13], [14], [15]. Despite the distinct outcome, some of the signaling pathways are conserved in both cases. For example, the expression of the peroxisome proliferator-activated receptor γ coactivator 1α (PGC-1α) in humans and rodents is temporarily elevated after each exercise bout [16], [17], [18], and exhibits a persistent basal elevation after chronic endurance training [19], [20], [21] while maintaining additional inducibility with physical activity [22], [23]. The acute increase of PGC-1α levels and activity might primarily be responsible for increased oxidative metabolism and hence an elevated ATP synthesis [24], [25], [26]. Chronic elevation of PGC-1α is associated with a fiber-type switch from the glycolytic, fast-twitch type IIb and IIx fibers towards the oxidative, high-endurance type IIa and I muscle fibers [27], [28] as well as an increase in vascularization [29]. In fact, PGC-1α seems to regulate many, if not all of the adaptations of muscle fibers to chronic endurance training [3], [28], [30], [31], [32], [33], [34] and leads to improved exercise performance as well as increased peak oxygen uptake [35].

We aimed at establishing and validating an EPS condition that induces a gene expression pattern resembling that of a trained muscle. For that purpose, we tested EPS protocols in different muscle cell types, and because of the versatility and robustness of PGC-1α induction in the trained muscle [36], we measured the expression of this gene as a prototypical exercise gene. Subsequently, the level of key genes in mitochondrial function, substrate uptake and oxidation were determined. The EPS-triggered changes of these genes in muscle fibers in culture were then compared to those in acutely exercised and trained mice, respectively. Our data suggest that the proper EPS conditions in muscle cells in culture qualitatively recapitulate some of the gene expression patterns observed in trained muscle.

## Results

### Electric pulse stimulation of C2C12 mouse muscle cells induces PGC-1α gene expression

**Figure 1:**
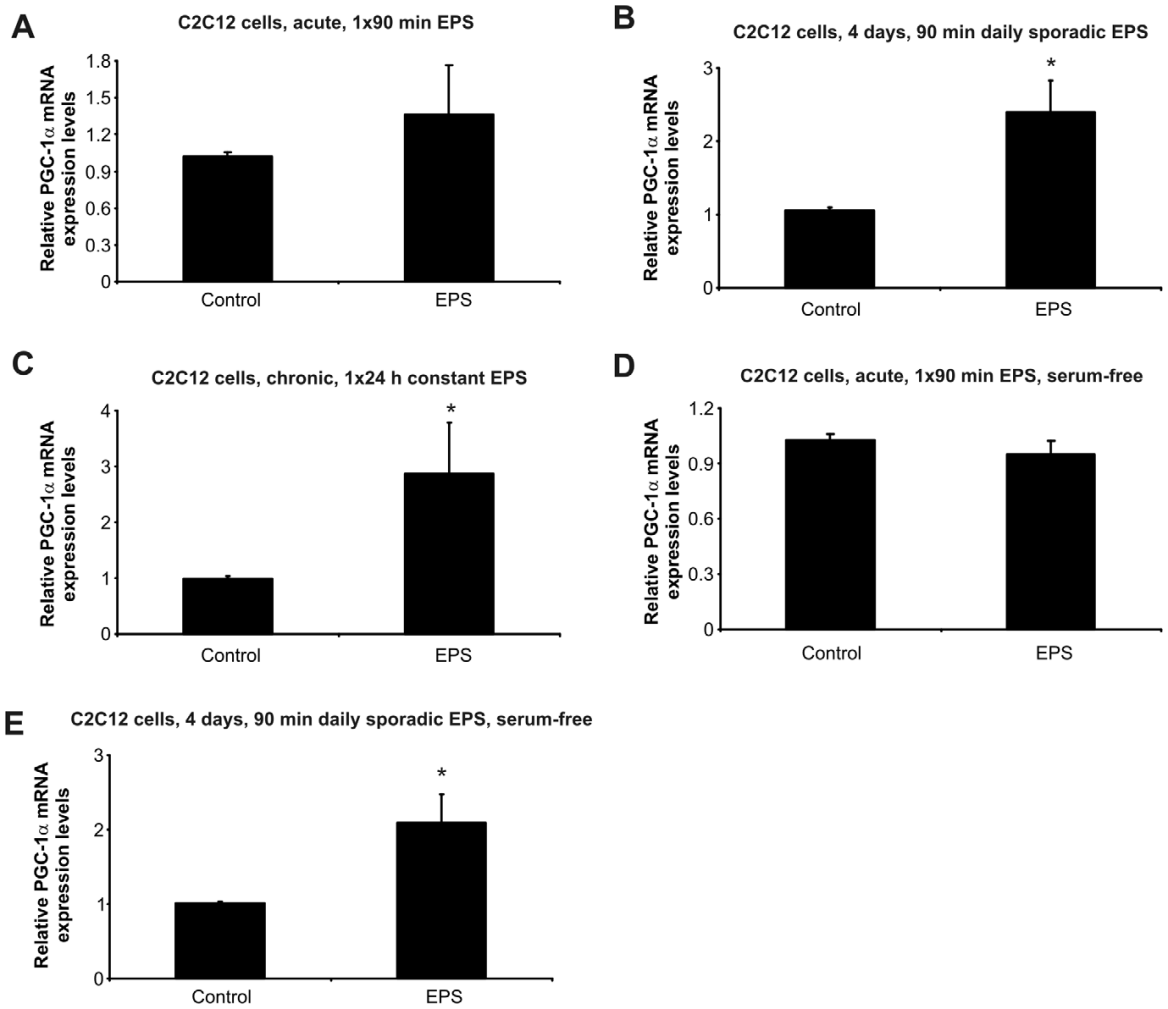
Electric pulse stimulation (EPS) of C2C12 myotubes induces PGC-1α gene expression. Differentiated C2C12 myotubes were electrically stimulated for 90 min (acute EPS, panels A and D), 4 consecutive days, 90 min each day (4 days, sporadic EPS, panels B and E) or for 24 h (24 h, chronic EPS, panel C). These experiments were either performed in regular differentiation medium (panels A, B and C) or in serum-free conditions with BSA (serum-free, panels D and E). Three hours after EPS, cells were harvested, RNA isolated and the relative expression of PGC-1α determined by real-time PCR. Bars are average levels of three independent experiments and error bars depict standard deviations. *p<0.05. Abbreviations: EPS, electric pulse stimulation.

Several different protocols for electric pulse stimulation (EPS) of muscle cells in culture have been proposed (e.g., see refs. [5], [6], [7], [8], [37], [38]). However, none of these approaches included a broad investigation of the transcriptional adaptations associated with an active muscle fiber. We thus tested two different EPS conditions and studied three different cell systems to establish stimulation conditions that recapitulate plastic changes on the gene expression level. As a marker for a trained muscle, we measured the relative expression of PGC-1α, a transcriptional coactivator that is regulated by endurance exercise in muscle and in turn controls many of the muscle adaptations to physical activity [32], [33], [34], [39]. For that purpose, differentiated C2C12 myotubes were either stimulated for 90 min (“acute”, Fig. 1A and D), for 90 min daily on 4 consecutive days (“intermittent”, Fig. 1B and E) or for 24 h (“continuous”, Fig. 1C). The cells were harvested 3 h after the EPS and the relative expression of PGC-1α was quantified by real-time PCR. A single stimulation for 90 min failed to alter PGC-1α expression significantly (Fig. 1A). In contrast, repeated EPS for 90 min daily on 4 consecutive days significantly induced PGC-1α gene expression (2.4 fold, Fig. 1B). Finally, a single EPS for 24 h resulted in the highest induction of PGC-1α transcript levels (2.9 fold, Fig. 1C). Silveira and colleagues demonstrated an enhanced reactive oxygen species (ROS) production in EPS-treated primary muscle cells and postulated an important role for elevated ROS levels in the induction of PGC-1α in these cells [8]. We therefore studied if EPS of C2C12 cells in serum-free and hence antioxidant-reduced conditions exacerbates PGC-1α induction. However, elevation of PGC-1α gene transcription was lower using serum-free compared to standard differentiation media (Fig. 1D, 2.1 fold) or did not reach statistical significance (Fig. 1E). When stimulated for 24 h in serum-free conditions, C2C12 cells changed in morphology and adhesion and therefore, gene expression changes were not assessed for this condition. The discrepancies between the previously reported findings and our data could be explained by the different culture conditions and cell types that were used in the previous study (primary rat muscle cells, ref. [8]) and in the present work (C2C12 muscle cells). To include additional mouse muscle culture models in our experiments, we tested the response of SOL8 cells, a myogenic cell line derived from mouse soleus primary cells, to the different EPS regimes and culture conditions. However, none of our stimulation protocols elicited a significant change in PGC-1α mRNA expression (data not shown). Whether this is due to the origin of SOL8 cells that were isolated from soleus, a muscle that shows a very modest response to endurance exercise, is unclear. Moreover, primary mouse muscle myotubes were overly sensitive to our EPS conditions and did not survive the respective experiments (data not shown). As a consequence of these tests, we used C2C12 cells and the 24 h EPS paradigm in all subsequent experiments.

### Improved exercise performance of mice after 6 weeks of endurance training

**Figure 2:**
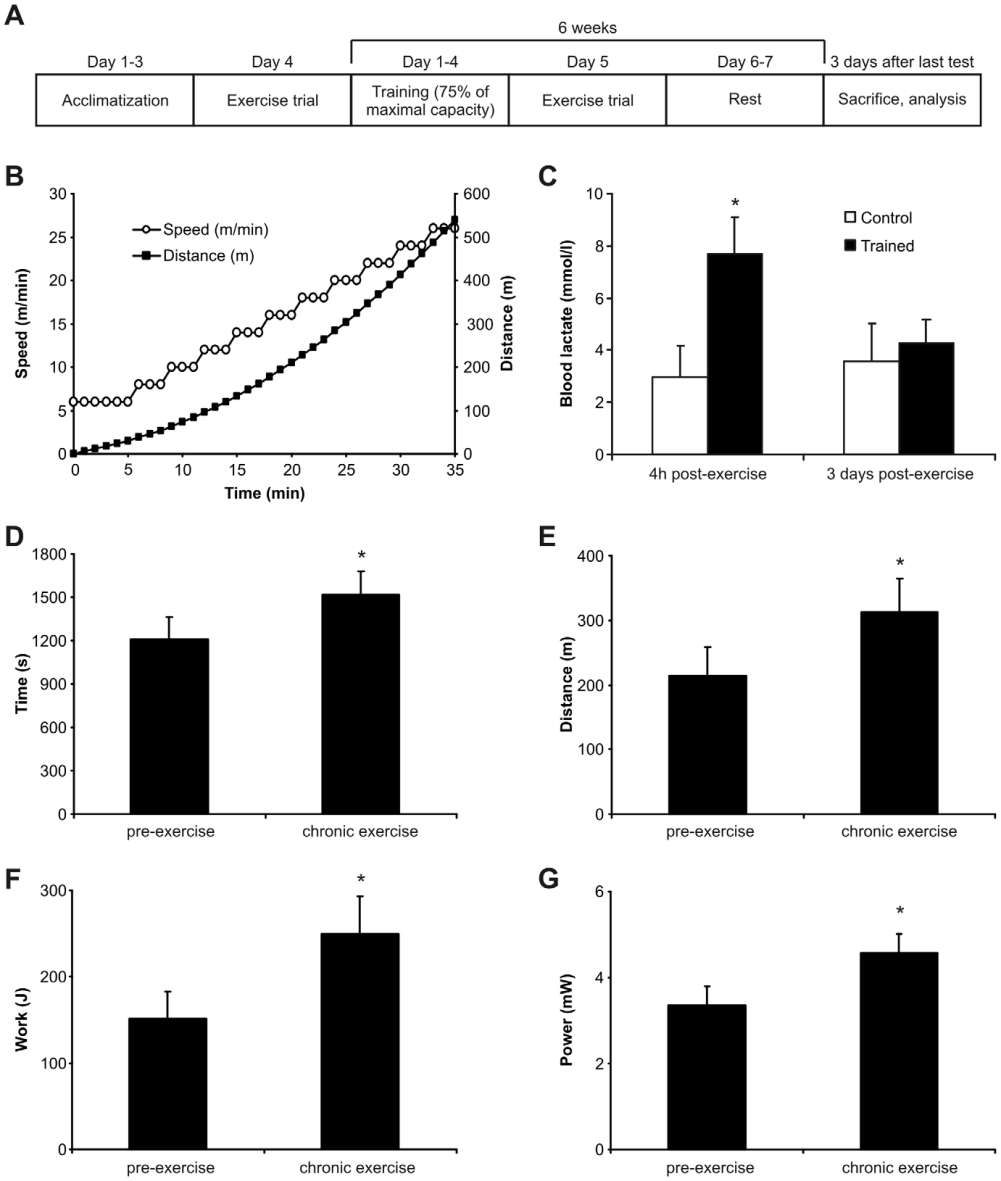
Endurance improvement of chronically exercised mice. **A,** After familiarization, mice were trained for 6 weeks and sacrificed 3 days after the last bout of exercise. This cohort (“chronic exercise”) was compared to a group of mice that performed one exercise trial (“acute exercise”) and sedentary controls. **B,** An exercise protocol with incremental speed was chosen for the endurance trial and the daily training. **C,** Blood lactate measurement in mice after an acute bout of exercise (4h post exercise) and after 3 days of recovery. **D–G,** Endurance running performance of mice before (pre-exercise) and after (chronic exercise) the 6 week training: running time (panel D), distance (panel E), work (panel F) and power (panel G). Bars are average levels and error bars depict standard deviations. N = 8 animals per group pre-exercise and n = 7 animals per group for the chronic exercise (one mouse died during the training period). *p<0.05.

Health benefits of exercise are primarily associated with training whereas a single exercise bout is insufficient to induce the same effects. To be able to compare the transcriptional changes of stimulated C2C12 cells in culture to those in acutely exercised and trained muscle, we designed an exercise protocol for mice that iteratively adjusts the training load to the increased muscle function over 6 weeks (Fig. 2A). This trained mouse cohort was then compared to a group of mice that underwent one endurance trial (called “mice, acute exercise”) and to control mice that remained sedentary, respectively. The endurance trial and the training bouts consisted of treadmill running with incremental speed (Fig. 2B), either to complete exhaustion for the trials or to 75% of the distance of the last exhaustion test for the training. In order to achieve a clear distinction between the acute effects of exercise and the chronic adaptations, the acute group was sacrificed 4 h after the trial when blood lactate levels were still elevated (Fig. 2C). In contrast, the trained group was analyzed 3 days after their last bout of exercise indicating an absence of confounding effects of the last acute exercise. At this time, the mice had normalized blood lactate (Fig. 2C). The improvement of endurance exercise capacity in the trained animals was documented by significant increases in time on the treadmill, distance, work and power after 6 weeks of training (Fig. 2D–G).

### Induction of mitochondrial regulators and genes by EPS

**Figure 3:**
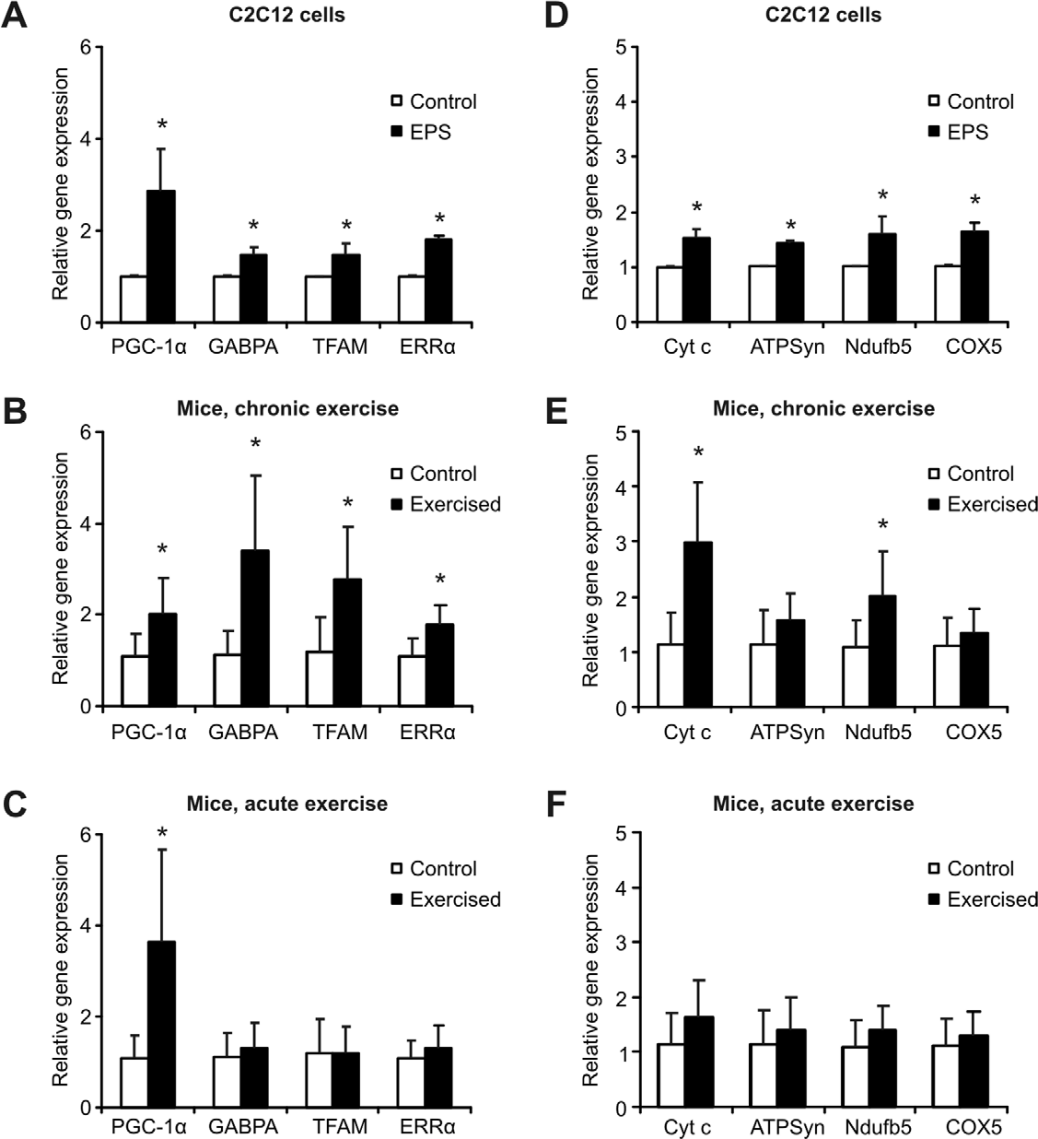
Electric pulse stimulation of C2C12 myotubes induces mitochondrial regulators and OXPHOS genes. **A, D,** C2C12 myotubes were EPS-stimulated for 24 consecutive hours. Three hours after stimulation, cells were harvested, RNA prepared and relative gene expression quantified by real-time PCR. The transcript levels were compared to unstimulated control cells. **B, E,** Mice were trained for 6 weeks. The animals were sacrificed 3 days after the last exercise bout, RNA isolated from gastrocnemius and relative gene expression determined by real-time PCR. Transcriptional induction was compared to the gene expression in sedentary control mice. **C, F,** Mice underwent one endurance trial and sacrificed 4 h later. RNA was isolated, relative gene expression quantified by real-time PCR and compared to that of sedentary control mice. Bars are average levels and error bars depict standard deviations. The data of the C2C12 study are from three independent experiments. In the animal studies, n = 8 animals per group were used except for the chronic exercise cohort with n = 7. *p<0.05. Abbreviations: PGC-1α, peroxisome proliferator-activated receptor γ coactivator 1α; GABPA, GA-binding protein; TFAM, mitochondrial transcription factor A; ERRα, estrogen-related receptor α; Cyt c, cytochrome c; ATPSyn, ATP synthase subunit 5o; Ndufb5, NADH-ubiquinone oxidoreductase 1β subunit 5; COX5, cytochrome c oxidase subunit 5b; EPS, electric pulse stimulation.

Endurance training results in an improvement of mitochondrial function and oxidative metabolism [1], [40], [41], [42], [43]. We thus tested how EPS of C2C12 cells affected some of the regulators of these processes and genes encoding oxidative phosphorylation (OXPHOS) proteins. In addition to PGC-1α, the GA-binding protein A (GABPA, alternatively called nuclear respiratory factor 2a or NRF2a), the mitochondrial transcription factor A (TFAM) and the estrogen-related receptor α (ERRα, NR3B1) were transcriptionally induced in stimulated cells compared to controls (2.9, 1.5, 1.5 and 1.8 fold, respectively, Fig. 3A). A similar expression pattern was observed in trained mice where PGC-1α was elevated 2.0 fold, GABPA 3.4 fold, TFAM 2.8 fold and ERRα 1.8 fold (Fig. 3B). In contrast, only PGC-1α transcription was significantly increased in acutely exercised animals (3.6 fold, Fig. 3C). ERRα, GABPA and TFAM regulate the transcription of nuclear- and mitochondrial-encoded mitochondrial genes, respectively [44]. PGC-1α coactivates ERRα and GABPA and thereby greatly enhances the transcriptional activity of these proteins [31], [45], [46], [47]. Finally, the PGC-1α gene itself and those encoding these three transcription factors are transcriptional targets for the PGC-1α protein [24], [47], [48], [49]. As a consequence of this biological switch, OXPHOS and other mitochondrial genes are induced. Accordingly, EPS-treated C2C12 myotubes expressed higher levels of cytochrome c (Cyt c, 1.5 fold, Fig. 3D), ATP synthase subunit 5o (ATPSyn, 1.5 fold), NADH-ubiquinone oxidoreductase 1β subcomplex 5 (Ndufb5, 1.6 fold) and cytochrome c oxidase subunit 5b (COX5, 1.7 fold). In mice that were trained for 6 weeks, only cytochrome c and NADH-ubiquinone oxidoreductase 1β subcomplex 5 expression levels were significantly altered compared to sedentary control animals (3.0 and 2.0 fold, respectively, Fig. 3E). Four hours after an acute bout of exercise, none of these OXPHOS genes were significantly modulated in muscle (Fig. 3F).

### Increased gene expression for substrate uptake and utilization in exercised muscle cells and trained muscle

**Figure 4:**
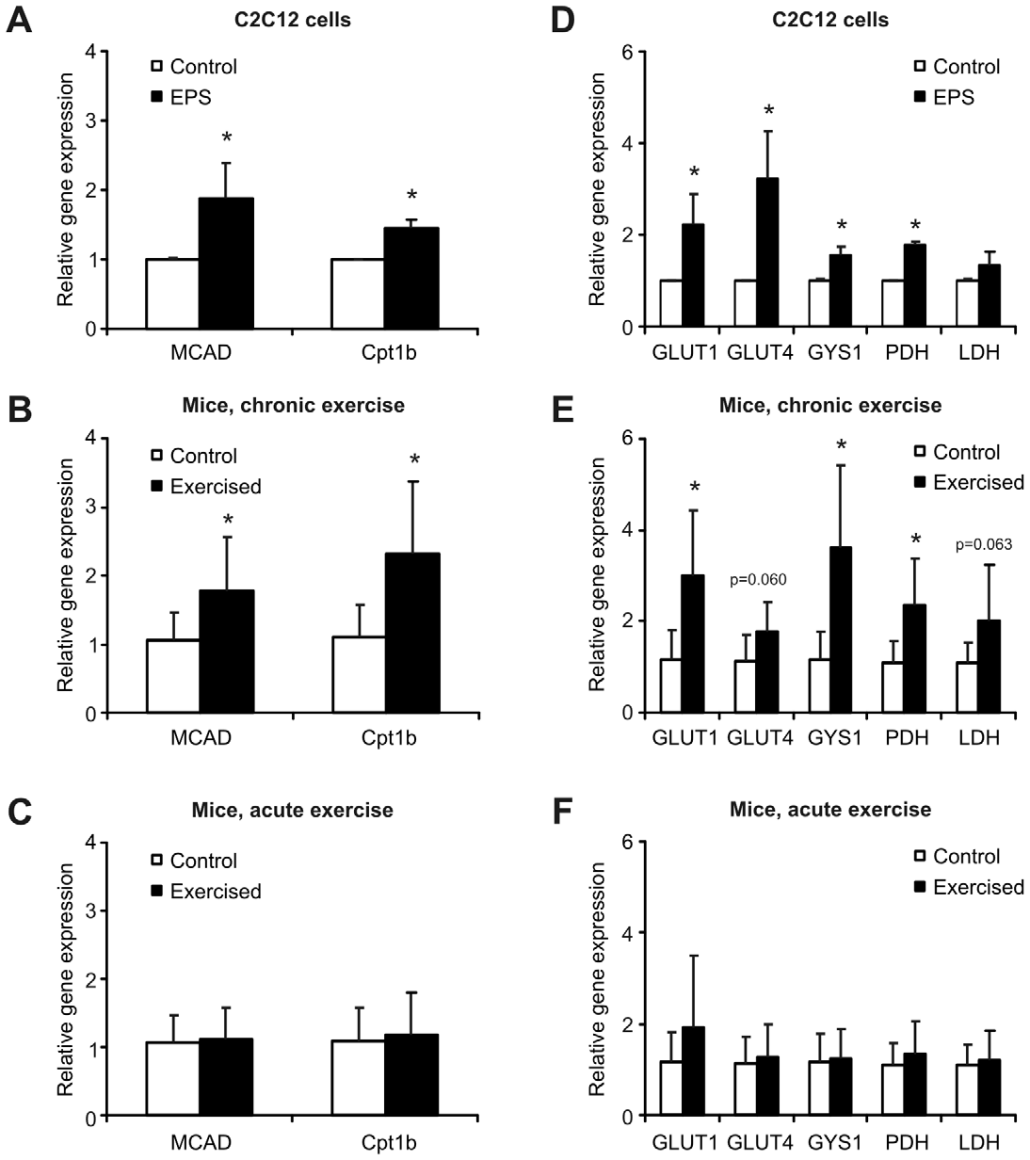
Electric pulse stimulation of C2C12 myotubes modulates fatty acid and glucose metabolism. **A, D,** C2C12 myotubes were EPS-stimulated for 24 consecutive hours. Three hours after stimulation, cells were harvested, RNA prepared and relative gene expression quantified by real-time PCR. The transcript levels were compared to unstimulated control cells. **B, E,** Mice were trained for 6 weeks. The animals were sacrificed 3 days after the last exercise bout, RNA isolated from gastrocnemius and relative gene expression determined by real-time PCR. Transcriptional induction was compared to the gene expression in sedentary control mice. **C, F,** Mice underwent one endurance trial and sacrificed 4 h later. RNA was isolated, relative gene expression quantified by real-time PCR and compared to that of sedentary control mice. Bars are average levels and error bars depict standard deviations. The data of the C2C12 study are from three independent experiments. In the animal studies, n = 8 animals per group were used except for the chronic exercise cohort with n = 7. *p<0.05. Abbreviations: MCAD, medium chain acyl-CoA dehydrogenase; Cpt1b, carnitine palmitoyltransferase 1b; GLUT1, glucose transporter 1; GLUT4, glucose transporter 4; GYS1, glycogen synthase 1; PDH, pyruvate dehydrogenase; LDH, lactate dehydrogenase; EPS, electric pulse stimulation.

The increase in mitochondrial OXPHOS genes in endurance-trained muscle allows a greater oxidative metabolism of substrates, both of glucose and fatty acids [50], [51]. Accordingly, we studied some of the genes encoding for key enzymes in substrate import and usage. Fatty acid β-oxidation is primarily regulated by the import of fatty acids into mitochondria. This step is mediated by the carnitine-palmitoyltransferase 1b (Cpt1b) in muscle [50], [52]. In the mitochondrial matrix, several proteins contribute to fatty acid oxidation, including the medium chain acyl-CoA dehydrogenase (MCAD) [50], [52]. Cpt-1b and MCAD expression was elevated in EPS-treated C2C12 myotubes compared to untreated cells (Fig. 4A, 1.9 fold and 1.4 fold, respectively). A similar increase in MCAD and Cpt1b expression was observed in trained mice (Fig. 4B, 1.8 and 2.3 fold, respectively) whereas no change in transcription was seen in acutely exercised animals (Fig. 4C). These data indicate that EPS of muscle cells in culture triggers an increase in fatty acid β-oxidation resembling the adaptations of this pathway in chronic endurance exercise.

In addition to fatty acids, trained fibers import and use more glucose than untrained muscle fibers [53], [54]. GLUT1 and GLUT4 are the major glucose transporters on the skeletal muscle cell membrane [55]. In the myocyte, glucose is primarily stored in the form of glycogen or alternatively channeled into the glycolytic pathway [55]. Subsequent to the glycolytic conversion to pyruvate, glucose is either metabolized to acetyl-CoA by the pyruvate dehydrogenase and fed into the Krebs cycle in the mitochondria for oxidative metabolism or reduced to lactate under anaerobic conditions by the lactate dehydrogenase. In order to gain insights about glucose metabolism in EPS-treated cells, we measured the expression of GLUT1, GLUT4, glycogen synthase 1 (GYS1), pyruvate dehydrogenase (PDH) and lactate dehydrogenase (LDH). In stimulated myotubes, GLUT1 (2.2 fold), GLUT4 (3.2 fold), glycogen synthase 1 (1.6 fold) and pyruvate dehydrogenase (1.8 fold) transcription were significantly elevated (Fig. 4D). In contrast, lactate dehydrogenase expression was unchanged (Fig. 4D). This gene expression pattern resembles that of trained mice where GLUT1 (3.0 fold), glycogen synthase 1 (3.6 fold) and pyruvate dehydrogenase (2.3 fold) were significantly induced (Fig. 4E). GLUT4 and lactate dehydrogenase gene expression changes failed to reach statistical significance (Fig. 4E). In contrast, none of the transcripts were altered in acutely exercised animals (Fig. 4F). In summary, our findings imply an increased import and oxidative metabolism of glucose as well as an elevated glycogen synthesis in EPS-treated C2C12 myotubes similar to the changes in trained muscle.

### Altered expression of myosin heavy chain isoforms in stimulated muscle cells

**Figure 5:**
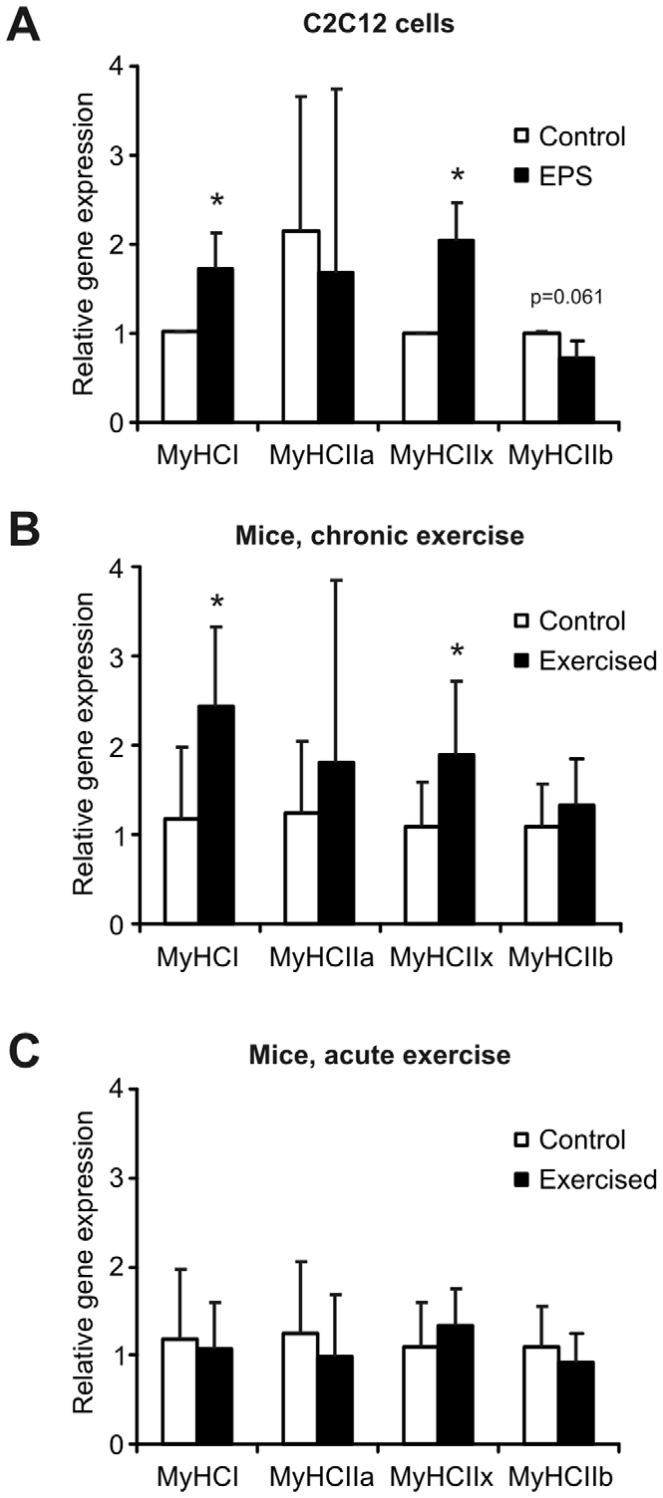
Electric pulse stimulation of C2C12 myotubes alters the myosin heavy chain expression patterns. **A,** C2C12 myotubes were EPS-stimulated for 24 consecutive hours. Three hours after stimulation, cells were harvested, RNA prepared and relative gene expression quantified by real-time PCR. The transcript levels were compared to unstimulated control cells. **B,** Mice were trained for 6 weeks. The animals were sacrificed 3 days after the last exercise bout, RNA isolated from gastrocnemius and relative gene expression determined by real-time PCR. Transcriptional induction was compared to the gene expression in sedentary control mice. **C,** Mice underwent one endurance trial and sacrificed 4 h later. RNA was isolated, relative gene expression quantified by real-time PCR and compared to that of sedentary control mice. Bars are average levels and error bars depict standard deviations. The data of the C2C12 study are from three independent experiments. In the animal studies, n = 8 animals per group were used except for the chronic exercise cohort with n = 7. *p<0.05. Abbreviations: MyHCI, myosin heavy chain I; MyHCIIa, myosin heavy chain IIa; MyHCIIx, myosin heavy chain IIx; MyHCIIb, myosin heavy chain IIb; EPS, electric pulse stimulation.

Skeletal muscle plasticity following endurance exercise extends beyond metabolic gene expression. For example, a close association between oxidative capacity and the levels of the myosin heavy chains I and IIa has been reported [56], [57], [58]. These oxidative, high endurance muscle fibers are classified as type I and IIa fibers [56], [57], [58]. In contrast, the type IIx and IIb muscle fibers are characterized by a fast twitching that generates a high force and express the myosin heavy chain isoforms IIx and IIb [56], [57], [58]. We studied if our EPS protocol can induce expression changes of genes other than those that are directly involved in substrate metabolism and therefore quantified the relative transcript levels of the four main myosin heavy chain isoforms expressed in the adult muscle. These experiments revealed a significant elevation of the expression of myosin heavy chain I (MyHCI, 1.7 fold) and myosin heavy chain IIx (MyHCIIx, 2.0 fold), whereas transcription of myosin heavy chain IIa and IIb (MyHCIIa and MyHCIIb) was unchanged (Fig. 5A). Our chronic exercise paradigm elicited similar changes *in vivo*. In these mice, myosin heavy chains I and IIx transcript levels were upregulated (2.4 and 1.9 fold, respectively) while the expression of myosin heavy chains IIa and IIb was unchanged (Fig. 5B). In muscle tissue of acute exercise mice, none of the myosin heavy chains exhibited altered expression levels (Fig. 5C). Thus, EPS of C2C12 myotubes triggered a modulation of myofibrillar gene expression resembling that of trained muscle.

### Changes in protein levels in EPS-treated C2C12 myotubes

**Figure 6:**
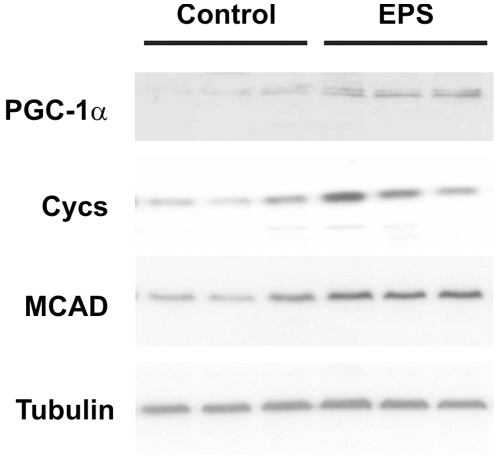
Elevation of protein levels in EPS-treated C2C12 myotubes. Protein extracts of control and EPS-treated C2C12 myotubes were analyzed in Western blots for the expression of PGC-1α, cytochrome c (Cycs), medium chain acyl-CoA dehydrogenase (MCAD) and tubulin.

The relative changes in transcript levels and those observed for the respective protein can differ significantly, Thus, to test whether the EPS treatment of C2C12 myotubes also alters protein levels in addition to the induction in gene expression, we performed Western blots using antibodies for a selection of the genes that were analyzed by real-time PCR (Fig. 6). We observed higher protein levels for PGC-1α, cytochrome c (Cycs) and the medium chain acyl-CoA dehydrogenase (MCAD) (Fig. 6) that reflect the higher transcript levels of the respective genes (Fig. 3A, 3D and 4A, respectively).

## Discussion

Exercise results in extensive adaptations in skeletal muscle and other organs [3], [14], [43]. Due to this complexity, our understanding of the molecular processes in these plastic changes is rudimentary. Furthermore, experimentally amenable model systems for exercise remain elusive. In this report, we describe how a cell culture model can be established to study at least some of the gene expression changes in the trained muscle. We found that key genes in metabolic processes, substrate uptake and oxidation are regulated by EPS in these cells. The pattern of gene expression qualitatively resembles very closely that of trained, but not acutely exercised muscle. Importantly, adaptation of muscle to training confers health benefits much more than the acute changes following a single bout of exercise.

Our analysis centered on the expression and function of the coactivator PGC-1α. This gene is central to the adaptations of muscle to exercise by transcriptionally regulating this biological program. Upon ectopic expression with viral vectors, PGC-1α induces and coactivates some of its binding partners [47]. Here, we demonstrate that EPS-triggered elevation of PGC-1α is likewise sufficient to increase the levels of these transcription factors and subsequently, of mitochondrial OXPHOS genes. Moreover, the relative expression levels of genes encoding fatty acid import and β-oxidation proteins and those involved in glucose uptake and conversion of pyruvate to acetyl-CoA suggest that concomitant with an elevated mitochondrial function, substrate availability and metabolism are increased in EPS-treated C2C12 cells and trained muscle. Finally, the elevated expression of the glycogen synthase in EPS-treated cells mirrors that of glycogen synthase in exercise *in vivo*. In the latter, augmented glycogen synthesis helps to replenish and expand the glycogen storage in trained muscle [59], [60]. Thus, the gene expression pattern in EPS-treated C2C12 myotubes suggests an extensive metabolic remodeling as found in trained muscle.

While the metabolic gene expression determines substrate usage, other muscle fiber parameters, such as fiber contraction speed and peak force generation, are controlled by myofibrillar proteins. The respective gene expression of the myosin heavy chain isoforms is used to classify the fibers into different fiber-types [61]. The relative proportion of these fiber-types is to a large extent regulated by the motor neuron input [62], [63], although additional factors including genetic predisposition and imprinting exist [64], [65], [66]. The lack of innervation of muscle cells in culture is likely to contribute to the poorly attributable fiber-type of many muscle cell lines. Electric stimulation can at least partially substitute for the missing motor neurons. We have observed an increase in the expression of specific myosin heavy chains in EPS-treated C2C12 myotubes. Our data suggest that with an adjusted protocol, e.g. slow, continuous stimulation or fast, sporadic stimulation, certain fiber-types can be promoted in these cells in culture. Moreover, in combination with other approaches such as addition of slow or fast muscle extracts [67] or continuous mild heat stress [68], EPS of muscle cells in culture might be used to study fiber-type-specific properties.

Some of the previous studies on EPS in muscle cells in culture indicated that serum-free conditions might be better in inducing exercise-like adaptations compared to cells that are cultured with serum [8]. It is possible that the elevated levels in reactive oxygen species in serum-free medium contributes to EPS-mediated induction of muscle gene expression [8]. Interestingly, a similar negative effect of administration of large doses of antioxidant vitamins on exercise performance in humans has been shown [69]. In our experiments, no significant difference between serum-containing and serum-free conditions was observed, which could be explained by differences in culture and EPS conditions. Nevertheless, these findings indicate that EPS-treated C2C12 cells might be used to study the effects of reactive oxygen species and antioxidants, respectively, on muscle in exercise. Moreover, it might be interesting to use a known composite serum for these experiments, which then can be supplemented with additional factors and study their effect on exercise-like adaptation.

In summary, we have established an experimental protocol to qualitatively simulate some of the exercise-induced changes in gene expression in muscle cells in culture, at least for the genes that were included in the study. Clearly, a single bout of stimulation of cell in culture does not reflect the complexity and time-dependent adaptation of skeletal muscle to exercise *in vivo*. Besides the missing neuronal activation, other stimuli of training adaptation, e.g. the hormonal milieu, are missing. Second, the determination of protein levels, enzymatic activates and functional read-outs might differ between the cells in culture and muscle *in vivo*. We tested some of the proteins for which increased gene expression was observed in the EPS-treatment and found elevated protein levels for PGC-1α, Cycs and MCAD (Fig. 6). In any case, such an experimental system allows the delineation of molecular mechanisms important for the contracting muscle fiber that cannot be performed in muscle *in vivo*. In the future, this model system will have to be adapted and refined. In particular, combining EPS with mechanical stretch or temporary hypoxia might further help to approximate the environment that a fiber of a trained muscle is exposed to. Similarly, co-cultures of motor neurons with these muscle cells might improve the comparability of the culture systems with muscle *in vivo*. These interventions might increase the activation of the different signaling pathways that converge on PGC-1α in the exercised muscle [70] and thereby further improve this cell culture model system. This will be tested by quantifying the activation of these pathways, e.g., phosphorylation of the AMP-dependent protein kinase and its cellular targets. Ultimately, these cells might constitute a key tool to further elucidate the molecular mechanisms of muscle adaptation to activity and inactivity and test novel approaches to treat fiber atrophy and muscular dystrophies.

## Materials and Methods

### Chemicals and cell culture reagents

Dulbecco's modified Eagle's medium (DMEM), fetal bovine serum (FBS), phosphate-buffered saline (PBS, calcium- and magnesium-free), 1× trypsin-EDTA, penicillin-streptomycin (P/S), and horse serum (HS) were purchased from Invitrogen (Basel, Switzerland). Bovine serum albumin (BSA) was obtained from Sigma (Buchs, Switzerland). SYBR-green was purchased from Sigma (Buchs, Switzerland). Trizol, reagents for DNase digestion and reverse transcription were obtained from Invitrogen (Basel, Switzerland). Real-time PCR primers were synthesized by Microsynth (Balgach, Switzerland).

### Cell culture conditions

C2C12 (CRL-1772) and SOL8 (CRL-2174) cells were obtained from the American Type Culture Collection (ATCC) and cultured at 37°C in 5% CO_2_ on 100-mm plastic dishes containing culture medium. These cell lines are standard mouse muscle cells that were obtained from glycolytic and oxidative muscles, respectively, and that are widely used as a cell culture model for muscle in culture. Culture medium consisted of DMEM supplemented with 1% P/S and 10% FBS for C2C12 and 20% for SOL8, respectively. For the experiments, the cells were distributed onto 6 well plates (Corning, New York, USA). Upon reaching about 90% of cell confluence, the culture medium was switched to differentiation medium consisting of DMEM supplemented with 1% penicillin/streptomycin and 2% horse serum. This differentiation medium was changed every 24 h. After 4–6 days, differentiation into myotubes was completed and the cells used for EPS.

### Electric pulse stimulation (EPS)

EPS was applied to myotubes with a C-Pace EP culture pacer (IonOptix, Dublin, Ireland). This instrument emits bipolar pulses to the carbon electrodes of the C-dish with the electrodes hanging in the cell culture media. The contraction paradigm consisted of 1-s trains with 1-s pauses between the trains. During the 1-s trains, a 1-ms pulse stimulus with 14 V and a frequency of 50 Hz was applied. These 1-ms pulses most likely result in a combined activation of action potentials and the dihydropyridine receptor system [71]. The contractions of the myotubes were verified by examination under microscope. C2C12 and SOL8 cells were EPS treated either for 90 min (“acute” protocol), for 90 min each on 4 consecutive days at regular time intervals (“intermittent” protocol) or for 24 consecutive h (“continuous” protocol). Differentiation medium was exchanged 1 h before the stimulation was started. In order to test serum-free conditions, BSA medium (DMEM supplemented with 1% P/S and 0.1% BSA) was added to 3 wells of C2C12 cells instead of differentiation medium on the third and fourth day of stimulation or for the 24 h of stimulation. Cells were harvested in 1 ml Trizol 3 h after EPS similar to previous studies [8].

### Animal experiments

All procedures were approved by the Veterinary office of the canton of Zurich and performed following institutional guidelines. Male wild-type C57Bl/6J mice (age: 6 weeks, body weight: between 20 and 25g) were maintained in cages with a 12h∶12h light-dark cycle starting at 6:00 in a room at 22°C. Food and water were available *ad libitum*. The mice were randomly divided into experimental groups with seven (chronic) or eight (acute) mice per group. Exercise training was performed at the same time each day during the light cycle. At the end of the experiment, mice were sacrificed by CO_2_ inhalation, blood was collected by cardiac puncture and death ensured by cervical dislocation.

### Treadmill exercise

Mice were first familiarized with treadmill running for 3 days. The treadmill (Columbus Instruments, Ohio, USA) was equipped with an electric stimulation grid at the rear. The duration of these familiarization runs was 5 min with a speed of 6 m/min and an incline of +5°. On the day after familiarization, body weights were measured and a forced incremental exercise test to exhaustion was performed. This test was started with an incline (α) of +5° and a speed of 6 m/min for 5 min. After this initial phase, the speed was progressively increased by 2 m/min every 3 min. Animals ran until exhaustion, which was defined as the inability to remain on the treadmill despite repeated contact with the electric grid (0.4 mA) and mechanical prodding for more than 10 s. Once exhaustion was reached, the power of the shock grid was turned off. Running time was measured and running distance, work and power were calculated. Distance is a function of time and speed (Distance = v_treadmill_*t). Work is the product of body mass, gravity, vertical speed and time (Work = m_mouse_*g*v_treadmill_*tan[α]*t). Power is calculated as the product of body mass, gravity and vertical speed (Power = m_mouse_*g*v_treadmill_*tan[α]). The group that underwent training recovered for 2 days. Subsequently, these mice trained using the same treadmill protocol until 75% of the average distance of all the mice from the last exhaustion trial was reached. This training was performed on 4 days of the week, while the 5^th^ day was used for a new exhaustion test. This procedure (4 days training, 1 day exhaustion trial and 2 days of rest) was repeated for a total of 6 weeks when the gains in endurance parameters were significantly increased over those of control mice for two weeks in a row. The control group was kept at standard, sedentary conditions. All the mice were anesthetized with isofluran (Provet, Lyssach, Switzerland) either 4 h after the single performance trial for the acute exercise group or 3 days after their last endurance trial for the chronic exercise group. Animals were sacrificed by CO_2_ inhalation, blood was collected by cardiac puncture before death was ensured by cervical dislocation.

### Gene expression analysis and Western blots

Mice were dissected and m. gastrocnemius was prepared. The frozen gastrocnemius was pulverized and RNA prepared using Trizol reagent according to the manufacturer's instructions (Invitrogen). C2C12 and SOL8 cells were directly lyzed in Trizol reagent and subsequently processed. The purity of the resulting RNA was assessed by the 260 nm/280 nm ratio. Subsequently, DNase I digestion was performed and 1 µg of total RNA was reverse transcribed. Relative gene expression levels were determined by real time polymerase chain reaction using SYBR-green on an Mx3000P QPCR System (Stratagene, La Jolla, USA) according to the ΔΔCt method. Relative gene expression was normalized to 18S rRNA and TATA box binding protein (TBP) levels. The following primers were used: PGC-1α (forward TGATGTGAATGACTTGGATACAGACA, reverse GCTCATTGTTGTACTGGTTGGATATG), GABPA (forward CCAAGCACATTACGACCATTT, reverse CCGTGGACCAGCGTATAGGA), TFAM (forward CCGAAGTGTTTTTCCAGCAT, reverse CAGGGCTGCAATTTTCCTAA), ERRα (forward AGCAAGCCCCGATGGA, reverse GAGAAGCCTGGGATGCTCTT), Cyt c (forward GCAAGCATAAGACTGGACCAAA, reverse TTGTTGGCATCTGTGTAAGAGAATC), ATPSyn (forward AGGCCCTTTGCCAAGCTT, reverse TTCTCCTTAGATGCAGCAGAGTACA), Ndufb5 (forward TTTTCTCACGCGGAGCTTTC, reverse ATAAAGAAGGCTTGACGACAAACA), COX5 (forward GCTGCATCTGTGAAGAGGACAAC, reverse CAGCTTGTAATGGGTTCCACAGT), MCAD (forward AACACTTACTATGCCTCGATTGCA, reverse CCATAGCCTCCGAAAATCTGAA), Cpt1b (forward ATCATGTATCGCCGCAAACT, reverse CCATCTGGTAGGAGCACATGG), GLUT1 (forward CGAGGGACAGCCGATGTG, reverse TGCCGACCCTCTTCTTTCAT), GLUT4 (forward GATGAGAAACGGAAGTTGGAGAGA, reverse GCACCACTGCGATGATCAGA), GYS1 (forward GAACGCAGTGCTTTTCGAGG , reverse CCAGATAGTAGTTGTCACCCCAT), PDH (forward GAAGGCCCTGCATTCAACTTC, reverse ATAGGGACATCAGCACCAGTGA), LDH (forward GGAAGGAGGTTCACAAGCAG, reverse TCACAACATCCGAGATTCCA), MyHCI (forward CCTTGGCACCAATGTCCCGGCTC, reverse GAAGCGCAATGCAGAGTCGGTG), MyHCIIa (forward ATGAGCTCCGACGCCGAG, reverse TCTGTTAGCATGAACTGGTAGGCG), MyHCIIx (forward AAGGAGCAGGACACCAGCGCCCA, reverse ATCTCTTTGGTCACTTTCCTGCT), MyHCIIb (forward GTGATTTCTCCTGTCACCTCTC, reverse GGAGGACCGCAAGAACGTGCTGA), 18S rRNA (forward AGTCCCTGCCCTTTGTACACA, reverse CGATCCGAGGGCCTCACTA), TBP (forward GGCCTCTCAGAAGCATCACTA, reverse GCCAAGCCCTGAGCATAA).

Western blots were performed on protein extracts from C2C12 cells. Antibodies were purchased from Santa Cruz (PGC-1α, MCAD) and Cell Signaling (Cycs, tubulin), respectively.

### Lactate determination

Circulating lactate concentration in venous blood was measured 4 h and 3 days after exercise in the acute and the chronic exercise group, respectively. Lactate analysis was performed using a Biosen C_line (EKF-diagnostic, Barleben-Magdeburg, Germany).

### Statistical analysis

Results are presented as mean while error bars depict standard deviation (SD). Unpaired, two-tailed student's t test was performed, statistical significance was defined as p<0.05 and indicated by an asterisk. Eight mice per group, except for the trained cohort with seven animals, or three independent cell experiments were used.
